# Two Methods for Increased Specificity and Sensitivity in Loop-Mediated Isothermal Amplification

**DOI:** 10.3390/molecules20046048

**Published:** 2015-04-07

**Authors:** De-Guo Wang, Jeffrey D. Brewster, Moushumi Paul, Peggy M. Tomasula

**Affiliations:** 1 Henan Postdoctoral Research Base, Food and Bioengineering College, Xuchang University, Xuchang 461000, China; 2 Eastern Regional Research Center, Agricultural Research Service, United States Department of Agriculture, Wyndmoor, PA 19038, USA; E-Mails: Jeffrey.Brewster@ARS.USDA.GOV (J.D.B.); Moushumi.Paul@ARS.USDA.GOV (M.P.); Peggy.Tomasula@ARS.USDA.GOV (P.M.T.)

**Keywords:** loop-mediated isothermal amplification (LAMP), non-specific amplification, dimethyl sulfoxide (DMSO), Touchdown LAMP

## Abstract

The technique of loop-mediated isothermal amplification (LAMP) utilizes four (or six) primers targeting six (or eight) regions within a fairly small segment of a genome for amplification, with concentration higher than that used in traditional PCR methods. The high concentrations of primers used leads to an increased likelihood of non-specific amplification induced by primer dimers. In this study, a set of LAMP primers were designed targeting the *prf*A gene sequence of *Listeria monocytogenes*, and dimethyl sulfoxide (DMSO) as well as Touchdown LAMP were employed to increase the sensitivity and specificity of the LAMP reactions. The results indicate that the detection limit of this novel LAMP assay with the newly designed primers and additives was 10 fg per reaction, which is ten-fold more sensitive than a commercial Isothermal Amplification Kit and hundred-fold more sensitive than previously reported LAMP assays. This highly sensitive LAMP assay has been shown to detect 11 strains of *Listeria monocytogenes*, and does not detect other *Listeria* species (including *Listeria innocua* and *Listeria invanovii*), providing some advantages in specificity over commercial Isothermal Amplification Kits and previously reported LAMP assay.

## 1. Introduction

Loop-mediated isothermal amplification, developed and reported by Notomi *et al*., in 2000 [1], can specifically, sensitively and rapidly amplify nucleic acids with two pairs of primers recognizing 6 independent sequences of a target gene under isothermal conditions. Moreover, Nagamine *et al*., has advanced the method by putting forward loop primers that accelerate the LAMP reaction [2]. Therefore, the LAMP assay theoretically has the advantage of specificity, selectivity, and rapidity over polymerase chain reaction (PCR) [3,4], nucleic acid sequence based amplification (NASBA) [5,6], strand displacement amplification (SDA) [7], rolling circle amplification (RCA) [8], helicase dependent amplification (HDA) [9], and cross-priming amplification assay (CPA) [10,11]. For practical application of LAMP as well as reduction of the rate of false positive results in LAMP reactions, most researches currently focus on development of closed-tube detection to reduce aerosol pollution and cross pollution, which include the use of turbidity [12], SYBR Green I [13], PicoGreen [14], GelRed^TM^ [15,16], lateral flow dipstick [17], hydroxynaphthol blue dye [18], malachite green [19], microfluidic chips and GMR sensors as well as calcein used by Eiken Chemical Co., Ltd [20,21]. However, there is still no report studying non-specific amplification and cause of false positive results in LAMP reactions at present.

The objective of this paper is to study the cause and limit the rate of false positive results in LAMP reactions targeting *L. monocytogenes* as well as to increase the specificity and sensitivity of these LAMP reactions using DMSO and Touchdown LAMP.

## 2. Results and Discussion

### 2.1. Non-Specific Amplification of LAMP Primers Targeting hlyA of L. Monocytogenes

Although there were only two primers, non-specific amplification occurred in the isothermal amplification of four primer combinations out of seven combinations, and it was more obvious in the reaction of three primer combinations (*hly*A-FIP + *hly*A-LF, *hly*A-FIP + *hly*A-LB, *hly*A-FIP + *hly*A-B3), as Table 1 shown. Analysis on the three combinations indicated that they had the common situation that 3–4 bases at 3' end of both primers had two complementary sequences on a same primer, and such situation had been avoided when LAMP primers for *prf*A of *L. monocytogenes* were designed and screened in this study. Moreover, it was proved by the experiment that non-specific amplification caused by primer dimers was one reason for false positive results of LAMP.

**Table 1:** Non-specific amplification of varying loop-mediated isothermal amplification (LAMP) primer combination.

Results of Amplification	*hly*A-FIP + *hly*A-LF	*hly*A-FIP + *hly*A-LB	*hly*A-FIP + *hly*A-F3	*hly*A-FIP + *hly*A-B3	*hly*A-BIP + *hly*A-F3	*hly*A-BIP + *hly*A-LF	*hly*A-F3 + *hly*A-B3
Non-specific Amplification	4/4	4/4	1/4	4/4	0/4	0/4	0/4

### 2.2. Optimization of DMSO Concentration

**Figure 1:**
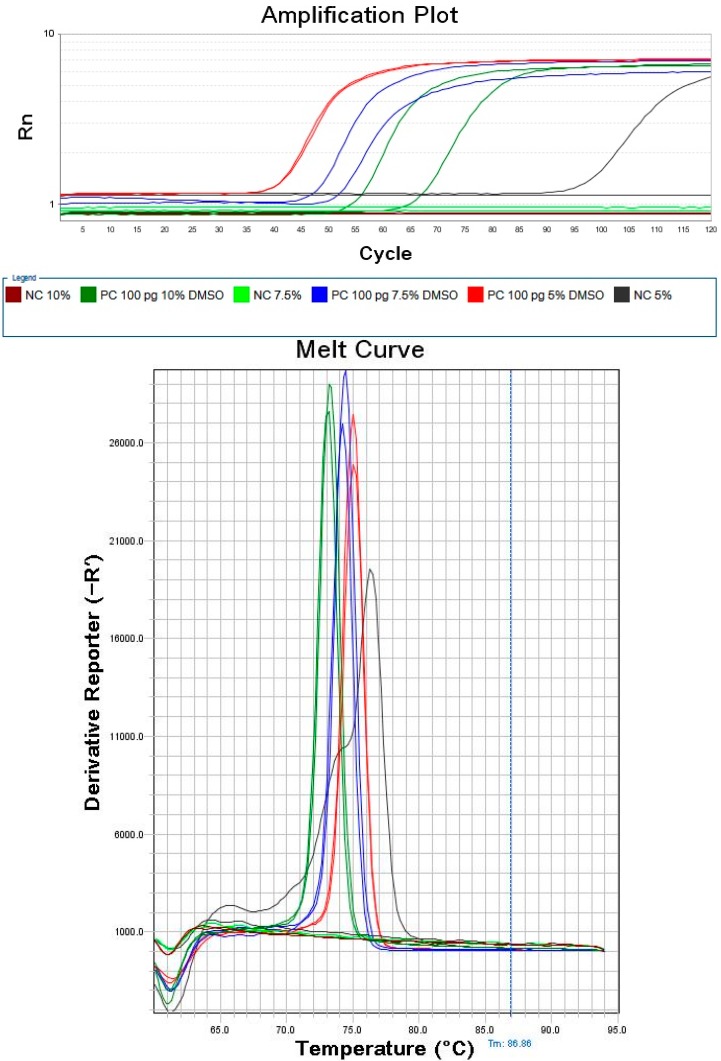
Amplification plot and melt curve of LAMP with varying concentrations of DMSO. The plot type was Rn *vs.* Cycle, and the graph type was log with 1× SYBR Green I added and 1× ROX as Reference Dye; the plot of Melt Curve was Derivative Reporter.

The LAMP reaction mixtures containing varying concentrations of DMSO were heated at 57 °C for 60 min (30 s per cycle), as indicated in Figure 1. When 5% DMSO was added, the detection time for 1 pg *L. monocytogenes* genomic DNA was less than 25 min; however, one of the two negative controls amplified as well. The T_m_ value and melt curve were obviously different from those of the positive controls, and were therefore as attributed to non-specific amplification, which may be caused by partial complementation among primers of LAMP. The detection time with 10% DMSO was slightly longer than with 7.5%. Overall, the results showed that the lower concentration of DMSO does not inhibit non-specific amplification while the higher concentration of DMSO may inhibit the activity of Bst 2.0 WarmStart DNA polymerase, and therefore, 7.5% DMSO was determined to be the optimal among these three concentrations.

### 2.3. Selection of Reaction Temperature for LAMP

7.5% DMSO was added to LAMP reaction mixtures and the reactions were carried out at varying temperatures for 60 min, as shown in Table 2. With a reaction temperature of 61 °C, only one of two positive controls (1 pg *L. monocytogenes* genomic DNA) was detected. The threshold time obtained using a reaction temperature of 57 °C was shorter than that obtained using 53 °C reaction temperature and slightly shorter than that obtained using the other temperatures. Therefore, 57 °C was chosen as the most suitable reaction temperature.

**Table 2:** Non-specific amplification of varying LAMP primer combination.

PC/NC	Threshold Time at 61 °C (min)	Threshold Time at 59 °C (min)	Threshold Time at 57 °C (min)	Threshold Time at 55 °C (min)	Threshold Time at 53 °C (min)
Positive Control (1 pg DNA)	42	28	26	28	36
	undetermined	32	26	27	56
Negative Control	undetermined	undetermined	undetermined	undetermined	undetermined
	undetermined	undetermined	undetermined	undetermined	undetermined

### 2.4. Detection Limit Comparison

The optimized LAMP reaction conditions were used with the conventional LAMP methodology with a serial dilution of *L. monocytogenes* DNA template was and these mixtures were heated at 57 °C for 60 min. As shown in Table 3, the detection limit of the optimized reaction mixture using the conventional LAMP technique was found to be 1000 fg *L. monocytogenes* DNA template.

**Table 3:** Sensitivity determination with different LAMP methods.

Results of Amplification	Developed Conventional LAMP Assay ^1^	Developed Touchdown LAMP Assay ^1^	Reported LAMP Assay ^2^	Isothermal Master Mix ^3^	Loopamp^®^ *Listeria monocytogenes* Detection Kit ^4^
Detection Limit	1000 fg (3/3)	10 fg (2/3)	1000 fg (1/3)	100 fg (1/3)	100 fg (3/3)
Non-specific Amplification of Negative Control	0/5	0/5	1/4	0/5	0/5

^1^ The Conventional LAMP Assay and Touchdown LAMP Assay were the methods developed in this paper; ^2^ Reported LAMP Assay was the method of the reference [22]; ^3^ Isothermal Master Mix: Manufactured by OptiGene Limited, West Sussex, UK; ^4^ Loopamp^®^ *Listeria monocytogenes* Detection Kit: Manufactured by Eiken Chemical Co., Ltd., Tochigi, Japan.

The optimized LAMP mixture was used with the Touchdown methodology to detect a serial dilution of *L. monocytogenes* DNA template. After the mixtures were preheated at 95 °C for 5 min and Bst 2.0 WarmStart DNA polymerase (New England Biolabs, Beverly, MA, USA) were added, they were heated at 63 °C for 5 min, at 61 °C for 5 min, at 59 °C for 5 min and then at 57 °C for 60 min, and, as indicated in Table 3, the sensitivity of Touchdown LAMP was found to be 10 fg of *L. monocytogenes* DNA. Therefore comparing these identical reaction mixtures in the conventional LAMP assay and the Touchdown LAMP assay shows that the Touchdown LAMP method increases the overall sensitivity of LAMP assay.

The detection limit of the original reported LAMP method by Tang, *et al* (Tang method) tested using 1000 fg *L. monocytogenes* DNA template, as well. Only one of 3 positives controls amplified using these conditions. Moreover, one of four negative controls showed non-specific amplification, as reported in Table 3.

Sensitivity of both the Isothermal Master Mix using our own designed LAMP primers as well as the Loopamp^®^ *Listeria monocytogenes* Detection Kit to detect *L. monocytogenes* was tested. The results indicate that the detection limit of both commercial LAMP kits is 100 fg *L. monocytogenes* DNA template per reaction, as shown in Table 4.

Therefore, the sensitivity of the newly developed LAMP assay presented here was ten-fold higher than that obtained using the commercial Isothermal Amplification Kits and hundred-fold higher than the originally reported Tang LAMP assay.

### 2.5. Assaying Selectivity

This newly developed LAMP assay was tested with 11 *Listeria monocytogenes* strains (10 stereotypes) and as shown in Table 4, all 11 were successfully detected. The assay was also tested with five other *Listeria* species. In the initial experiment, *Listeria invanovii ATCC49954* was falsely detected and one of three reactions amplified, while the other species had negative results. The experiment with *L. invanovii* *ATCC49954* was repeated four times and all four repeated reactions were negative. Therefore, the initial false positive result may have been caused by slight aerosol pollution of DNA templates.

**Table 4:** Specificity determination with different LAMP methods.

Bacterial Strain (Serotype)	Developed Touchdown LAMP Assay ^1^	Reported LAMP Assay ^2^	Isothermal Master Mix ^3^	Loopamp^®^ *Listeria monocytogenes* Detection Kit ^4^
*Listeria monocytogenes* J1-225 (4b)	3/3	2/2	3/3	3/3
*Listeria monocytogenes* J2-020 (1/2a)	3/3	2/2	3/3	3/3
*Listeria monocytogenes* J2-064 (1/2b)	3/3	1/2	3/3	3/3
*Listeria monocytogenes* J1-169 (3b)	3/3	2/2	3/3	3/3
*Listeria monocytogenes* J1-049 (3c)	3/3	2/2	3/3	3/3
*Listeria monocytogenes* M1-004 (N/A)	3/3	2/2	3/3	3/3
*Listeria monocytogenes* J1-094 (1/2c)	3/3	2/2	3/3	3/3
*Listeria monocytogenes* C1-115 (3a)	3/3	2/2	3/3	3/3
*Listeria monocytogenes* J1-031 (4a)	3/3	2/2	5/7	3/3
*Listeria monocytogenes* W1-110 (4c)	3/3	2/2	3/3	3/3
*Listeria monocytogenes* ATCC19115 (4b)	3/3	1/2	3/3	3/3
*Listeria innocua* ATCC51742	0/3	0/2	0/3	1/7
*Listeria invanovii* ATCC49954	1/7	0/2	0/3	0/3
*Salmonella typhimuriam*	0/3	0/2	0/3	0/3
*Salmonella enterica* serotype Newport	0/3	0/2	0/3	0/3
*Escherichia coli* O157:H7 933	0/3	0/2	0/3	0/3
Negative Controls	0/7	0/3	1/6	1/8

^1^ The Conventional LAMP Assay and Touchdown LAMP Assay were the methods developed in this paper; ^2^ Reported LAMP Assay was the method of the reference [22]; ^3^ Isothermal Master Mix: Manufactured by OptiGene Limited; ^4^ Loopamp^®^ *Listeria monocytogenes* Detection Kit: Manufactured by Eiken Chemical Co., Ltd.

While the reported LAMP assay can favorably differentiate *Listeria monocytogenes* from other *Listeria* species, the detection time was quite long. The amplification time originally reported by Tang, *et al.* was 40 min [19]; however, the optimized LAMP assay required an extended amplification time of 50 min, and even with the lengthy reaction time, two strains (*Listeria monocytogenes* J2-064 (stereotype: 1/2b) and *Listeria monocytogenes* ATCC19115 (stereotype: 4b) were not detected, as shown in Table 4. Extending the amplification time further to 1 h, led to non-specific amplification of negative controls.

The two commercial LAMP Kits were able to distinguish *Listeria monocytogenes* from other *Listeria* species. There were, however, two negative controls that exhibited non-specific amplification, and, sometimes, *L. monocytogenes* J1-031 (stereotype: 4a) was not detected, as shown in Table 4.

Therefore, the newly developed LAMP assay presented here can detect 10 stereotypes of *Listeria monocytogenes* selectively while not detecting other *Listeria species* (including *Listeria innocua* and *Listeria invanovii*), and had some advantages over commercial Isothermal Amplification Kit and the original Tang LAMP assay in specificity.

## 3. Experimental Section

### 3.1. Primer Design

The LAMP primers targeting specific gene *hly*A of *Listeria monocytogenes* reported by Tang, *et al*., are used for studying non-specific amplification of LAMP [22], as shown in Table 5.

**Table 5:** Primers for detecting *prf*A of *L. monocytogenes* with LAMP.

Primer	Sequence (5'–3')
*hly*A-FIP	CGTGTTTCTTTTCGATTGGCGTCTTTTTTTCATCCATGGCACCACC
*hly*A-BIP	CCACGGAGATGCAGTGACAAATGTTTTGGATTTCTTCTTTTTCTCCACAAC
*hly*A-F3	TTGCGCAACAAACTGAAGC
*hly*A-B3	GCTTTTACGAGAGCACCTGG
*hly*A-LF	TAGGACTTGCAGGCGGAGATG
*hly*A-LB	GCCAAGAAAAGGTTACAAAGATGG
*prf*A-FIP	CCGCTCCTTTTTAATTCGTAAAACTTTTTAAAACGTGTCCTTAACTCTCTC
*prf*A-BIP	ATCATGGTAATAGCTTTTCAGGCTTTTTTTTGAAGTTTTTCTTCCCCG
*prf*A-F3	AACGTATAATTTAGTTCCCACAT
*prf*A-B3	GGGTCTTTTTGGCTTGTGTA
*prf*A-LF	TTAAGCCACCTACAACTAATCTGAC
*prf*A-LB	CATTTCACTATGACGGTAAAAGCAG

Targeting the specific gene *prf*A (GenBank Locus: AY512430.1) of *L. monocytogenes*, a set of LAMP primers were designed and selected with PrimerExplorer 4 and Oligo 7 according to the reported methodology [23], and are listed in Table 5.

### 3.2. Non-Specific Amplification of LAMP Primers

The isothermal amplification was performed in a total 25 μL reaction mixture containing 1.25 mM dNTPs, 1 M betaine (Sigma-Aldrich Corp, St Louis, MO, USA), 20 mM Tris-HCl (pH 8.8), 10 mM KCl, 10 mM (NH_4_)_2_SO_4_, 6 mM MgSO_4_, 0.1% Triton X-100 and 8 units of Bst 2.0 WarmStart DNA polymerase (New England Biolabs, Beverly, MA, USA) according to the report of Tang, *et al.* [22], different combination of LAMP primers targeting *hly*A were added into different tube, as listed in Table 2, the concentrations of *hly*A-FIP, *hly*A-BIP, *hly*A-F3, *hly*A-B3, *hly*A-LF, and *hly*A-LB were 0.8 mM, 0.8 mM, 0.4 mM, 0.4 mM, 0.1 mM and 0.1 mM, respectively, and DNA template was not added for determination of non-specific amplification. The amplification reaction was performed at 65 °C for 50 min in StepOne^TM^ System (Applied Biosystems, Foster City, CA, USA).

### 3.3. Optimization of DMSO Concentration

LAMP was performed in 10 μL reaction mixture containing 0.8 mM each of *prf*A-FIP and *prf*A-BIP, 0.2 mM each of *prf*A-F3 and *prf*A-B3, 0.4 mM each of *prf*A-LF and *prf*A-LB, 1.0 mM dNTPs, 20 mM Tris-HCl (pH 8.8), 10 mM KCl, 10 mM (NH_4_)_2_SO_4_, 6 mM MgSO_4_, 0.1% Triton X-100, 3.2 units of the Bst 2.0 WarmStart DNA polymerase (New England Biolabs, Beverly, MA, USA), 1× EvaGreen, 1× Rox, 1 pg *L. monocytogenes* DNA template [1,22]. 5%, 7.5% and 10% DMSO were added into different reaction tubes. LAMP was carried out at 57 °C for 60 min and a melt curve was obtained using a StepOne^TM^ System.

### 3.4. Selection of Reaction Temperature for LAMP

LAMP was performed as above in a 10 μL reaction mixture containing 1 pg *L. monocytogenes* DNA template as well as the optimized concentration DMSO at 61, 59, 57, 55 and 53 °C for 60 min, and a melt curve was obtained using a StepOne^TM^ System.

### 3.5. Sensitivity Comparison of Our Developed LAMP Assay, Reported LAMP Assay and Commercial Isothermal Amplification Kit

The optimized LAMP mixture was combined with serial dilutions of DNA template of *Listeria monocytogenes* ranging from 1 to 1000 fg, and the reaction mixtures were heated at selected temperature 57 °C for 60 min in StepOne^TM^ System, and the detection limit of conventional LAMP was determined.

In the case of Touchdown LAMP, the reaction mixture was preheated at 95 °C for 5 min. After 5 min, Bst 2.0 WarmStart DNA Polymerase (Large Fragment) was added and the reaction mixture was heated at temperatures 6 °C higher than selected temperature for 5 min, at temperature of 4 °C higher than selected temperature for 5 min, at temperature of 2 °C higher than selected temperature for 5 min and then at selected temperature for 60 min, and the sensitivity of Touchdown LAMP was determined and compared with that of conventional LAMP.

For comparison, the detection limit of the reported method by Tang, *et al*., (Tang LAMP assay) was determined by carrying out LAMP reactions according to the conditions specified in their publication [22].

Isothermal Master Mix and Loopamp^®^ *Listeria monocytogenes* Detection Kits were purchased from OptiGene Limited (West Sussex, UK) and Eiken Chemical Co., Ltd (Tochigi, Japan), respectively, and LAMP was carried out according to the manufacturers’ instructions using a set of serially diluted DNA template of *L. monocytogenes* ranging from 1 to 1000 fg.

### 3.6. Specificity Determination of Optimized Touchdown LAMP assay, Tang LAMP Assay and Commercial Isothermal Amplification Kit

Eleven strains of *L. monocytogenes* (different stereotype) and 5 other *Listeria* species (including *L. innocua* and *L. invanovii*) were used for the specificity study (Table 4). *Listeria* strains were cultured overnight at 37 °C in Difco^TM^ Buffered *Listeria* Enrichment Broth Base (Becton, Dickinson and Company, San Jose, CA, USA) and the others in Luria-Bertani (LB) broth. DNA from these pure cultures was extracted according to the manufacturer’s handbook of DNeasy^®^ Blood and Tissue Kit (QIAGEN LTD, North Manchester, UK), and these DNA templates was used for determining the specificity of the optimized Touchdown LAMP assay, the Tang LAMP assay and the LAMP assay utilizing the commercial Isothermal Amplification Kit. The amount of DNA template used is 1 pg per reaction.

## 4. Conclusions

It is difficult to avoid primer dimers and non-specific amplification when couple numerous sets of primers are used in LAMP assays. This is especially true when the concentrations of primers, Mg^2+^, dNTPs and DNA Polymerase in reaction mixtures are as high as those used in Real-time PCR. The concentrations of these 4 factors must be strictly controlled to avoid non-specific amplification in real-time PCR [24] as well as LAMP reactions. There are instances in which standard PCR amplification reaction conditions do not produce acceptable results. Addition of DMSO and use of Touchdown temperature conditions have been used improve PCR results. We investigate these approaches for the first time for optimization of LAMP reactions. Unfortunately, with the information presently available it is not possible to predict which enhancing agent is best for any particular target. But DMSO has been frequently used in this capacity [25]. The results presented here using DMSO in LAMP reaction mixtures indicate that, DMSO can increase amplification with LAMP at low concentration and can inhibit activity of Bst 2.0 WarmStart DNA polymerase. We had tried to enhance the reaction of LAMP with betaine, Tetramethylammonium chloride, tetramethylene sulfoxide, and formamide, but their effect was not as good as that of DMSO, because of the limitation of length, no more tautology here. While DMSO may not necessarily be the best enhancing agent [26,27], *i.e*., the manufacturer of the commercial Isothermal Amplification Kit used in this experiment may have found some favorable enhancing agent, but their reagents are proprietary, and DMSO served to decrease non-specific amplification in the specific experiments presented here.

Touchdown PCR offered a simple and rapid means to optimize PCRs, increasing specificity, sensitivity and yield, without the need for lengthy reaction times and/or the redesigning of primers [28,29]. Touchdown LAMP, compared to conventional LAMP methods, results in increased sensitivity and yield of LAMP. This improvement may be due to the high temperature inhibiting the formation of primer dimers and promoting the correct combination of primers and template. The biggest advantage of LAMP is that the reaction can be performed isothermally, and people argue all the time that all we need is a simple water bath for the rapid detection, so the advantage may be compromised by the developed Touchdown LAMP assay, we made such efforts here just to reveal or verify the main cause for false positive results of LAMP and inspire people to modify and improve LAMP technology, my colleagues and I have also been looking for a more suitable method, which can not only keep the advantage but also improve the sensitivity and specificity of LAMP.

In summary, non-specific amplification was a limiting factor in the applicability of the LAMP methodology. A few different options to eliminate this issue have been reported here to successfully selectively and sensitively detect *L. monocytogenes.* Designing ideal primers, additives such as DMSO, and method modifications such as Touchdown LAMP may be favorable alternatives for increased specificity and sensitivity in LAMP in other applications as well.
